# Diet quality impairs male and female reproductive performance and affects the opportunity for selection in an insect model

**DOI:** 10.1002/ece3.9533

**Published:** 2022-11-22

**Authors:** Lennart Winkler, Tim Janicke

**Affiliations:** ^1^ Applied Zoology TU Dresden Dresden Germany; ^2^ Centre d'Écologie Fonctionnelle et Évolutive CNRS, Univ Montpellier, EPHE, IRD Montpellier Cedex 05 France

**Keywords:** condition dependence, dietary restriction, environmental stress, opportunity for selection, sex‐specific selection, sexual selection

## Abstract

Environmental factors can have profound effects on the strength and direction of selection and recent studies suggest that such environment‐dependent selection can be sex‐specific. Sexual selection theory predicts that male fitness is more condition dependent compared to female fitness, suggesting that male fitness is more sensitive to environmental stress. However, our knowledge about the effect of environmental factors on sex‐specific reproductive performance and on sex differences in the opportunity for selection is still very limited. In the present study, we investigated the sex‐specific effects of diet quality (yeast deprivation and flour type) in the red flour beetle *Tribolium castaneum* . Specifically, we manipulated yeast supplementation in wheat and whole‐wheat flour in competition assays allowing us to test for sex‐specific effects of food quality (i) on reproductive success and (ii) on the opportunity for selection. Our data show that yeast deprivation in wheat flour had significantly negative effects on body mass and reproductive success of both sexes, while high‐quality flour (whole‐wheat flour) was able to buffer the negative impact to a large extent. Importantly, our data suggest no sex‐specific effect of dietary stress on reproductive success because the magnitude of the negative effect of yeast deprivation was similar for males and females. Moreover, our study demonstrates that low food quality inflated the opportunity for selection and did not differ between sexes neither under benign nor stressful dietary conditions. We discuss the implications of our findings for the adaptation to stressful environments.

## Graphical abstract

Environmental factors can have profound effects on the strength and direction of selection and recent studies suggest that such environmentdependent selection can be sex‐specific. We manipulated diet quality in an insect model finding no sex‐specific effect of dietary stress on reproductive success. Moreover, our study demonstrates that low food quality inflated the opportunity for selection and did not differ between sexes neither under benign nor stressful dietary conditions.

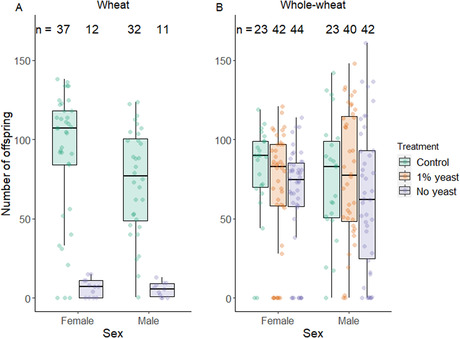

## 1 INTRODUCTION

Environmental factors have a profound impact on the demography of populations. They affect reproductive performance and can alter the strength and direction of selection (Riegl et al., 2017; Willi & Hoffmann, 2009). In general, stressful conditions are predicted to lower a population's growth (Riegl et al., 2017; Sommer et al., 2016; Willi & Hoffmann, 2009) and may impose stronger selection on deleterious alleles (Cally et al., 2019; Martinossi‐Allibert et al., 2019; Whitlock & Agrawal, 2009). Importantly, these effects can differ between males and females as a consequence of sex‐specific life‐history strategies (Berger et al., 2016; Janicke et al., 2015; Martinossi‐Allibert et al., 2017, 2019; Moiron et al., 2022). The postulated ‘live‐fast‐die‐young’ strategy of males implies that males invest more in current reproduction whereas females allocate more resources into future reproduction (Bonduriansky et al., 2008; Vinogradov, 1998; but see: Travers et al., 2015). Hence, females are expected to live longer (Viña et al., 2005), have a better immune competence (Kelly et al., 2018) and may also be more resilient when facing environmental stress. Moreover, environmental conditions often change the pool of resources that can be allocated to different fitness routes, also called conditions (Rowe & Houle, 1996). Sexual selection theory predicts that male reproductive performance is particularly condition‐dependent so that an unfavorable environment may have a stronger negative impact on males (Whitlock & Agrawal, 2009; Winkler et al., 2021). Despite the outlined theoretical framework predicting pervasive sex differences in stress response, the empirical evidence for sex‐specific effects of environmental factors is limited (but see e.g., Duxbury & Chapman, 2020; Reddiex et al., 2013).

Diet quality can severely influence an individual's reproductive success (Brooker et al., 2013; Duxbury & Chapman, 2020; Eldrigde & Krapu, 1988; García‐González et al., 2016; Geister et al., 2008; Katsuki et al., 2012; Naya et al., 2007). However, evidence for sex‐specific effects of diet quality is scarce. In the beetle *Gnatocerus cornutus*, diet quality has been shown to have sex‐specific fitness effects, with a stronger impact on males through larval nutritional environment and on females by adult diet (Katsuki et al., 2012). Moreover, in *Drosophila melanogaster* females benefited more than males from a high‐quality diet (Duxbury & Chapman, 2020), and also the black field cricket (*Teleogryllus commodus*) was found to show sex‐specific diet optima (Maklakov et al., 2008).

In the present study, we explore the effect of diet quality on the sex‐specific strength of selection in the red flour beetle *Tribolium castaneum*. To this end, we manipulated the diet by removing the baker's yeast (*Saccharomyces cerevisiae*) supplement from the flour mixture that forms the habitat of *T. castaneum*. Yeast supplementation could be important for red‐flour beetles, as it adds proteins (Schmidt et al., 1956) and nutritive supplements (James et al., 1987) to their diet. In addition, baker's yeast contains immunostimulating compounds (e.g. β‐glucans and nucleic acids) (Abdel‐Tawwab et al., 2008; Siwicki et al., 1994) that have been shown to increase fitness in several fish species (Abdel‐Tawwab et al., 2008; Ortuño et al., 2002; Sakai et al., 2001; Siwicki et al., 1994). Furthermore, lipids contained in a yeast‐enriched diet, have been found to influence membrane lipid composition and fitness in *D. melanogaster* (Brankatschk et al., 2014, 2018; Guo & Reinhardt, 2020). This is in line with previous studies in *T. castaneum* indicating that baker's yeast supplementation leads to shorter development time and increased productivity of populations (Sokoloff et al., 1966). Nevertheless, the effect of yeast availability on individual fitness and more importantly sex‐specific selection is unknown in *T. castaneum*. A previous study manipulated yeast availability for *T. castaneum* males and found that dietary restriction reduced spermatogenesis and testes size investment (Godwin et al., 2017). These findings suggest that yeast deprivation likely has a strong effect on male reproductive success.

Using two identical fitness assays, we studied the effect of yeast availability in both wheat flour and whole‐wheat flour on male and female reproductive success. While wheat flour is typically the standard laboratory food, whole‐wheat seems to be beneficial for development time (Chapman, 1924; Sokoloff et al., 1966; Wong & Lee, 2011) and overall fitness of *Tribolium* (Good, 1933; Park, 1934; Sokoloff et al., 1966; Wong & Lee, 2011), although these early studies focused on *T. confusum* and not *T. castaneum* (but see: Sokoloff et al., 1966; Wong & Lee, 2011). This could be due to the additional vitamins (i.e. vitamin E and folate), minerals and higher sugar content in whole‐wheat flour compared to wheat flour (Kumar et al., 2011; Likes et al., 2007). In the present study, we tested if the benefits of whole‐wheat flour can mitigate the negative impact of the removal of yeast from the diet.

We first evaluated the sex‐specific effects of diet quality on body mass as an indicator of condition. Secondly, we assessed the effect of diet on reproductive success using an assay in which stressed individuals are challenged by unstressed competitors. And finally, we estimated the sex‐specific opportunity for selection (measured as the variance in reproductive success [Crow, 1958; Wade & Shuster, 2005]) in populations of stressed and unstressed individuals. We hypothesize that diet has a stronger effect on reproductive success and on the opportunity for selection in males, due to the predicted higher condition dependence of male fitness (Janicke et al., 2016; Whitlock & Agrawal, 2009).

## 2 MATERIALS AND METHODS

Stock cultures and experimental cultures were kept in incubators at 30°C (±1°C) at 50% humidity (±5%) without light. Experimental procedures were performed at room temperature. All flour had been frozen for sterilization prior to experiments at −30°C for at least 24 h. The *Ga1* (wildtype line) and *Rd* (‘reindeer’ mutant line) used in this experiment had been originally supplied by the U.S. Department of Agriculture and kept in the laboratory for over a year in standard culturing conditions. *Rd* is a dominant mutation that affects antenna morphology and was used as a paternity marker. All stock beetles were kept in a flour mixture that consisted of organic wheat flour (type 405, Alnatura, Darmstadt, Germany) and 5% dry baker's yeast. In this study we manipulated the diet in two ways: First, we manipulated yeast availability in standard, organic wheat flour (type 405, Alnatura, Darmstadt, Germany), with either control (5% yeast) or no yeast. Secondly, we manipulated yeast availability in organic whole‐wheat flour (Alnatura, Darmstadt, Germany), with either control (5% yeast), low yeast availability (1% yeast) or no yeast. The low yeast availability treatment was with 1% yeast similar to a previous study (Godwin et al., 2017) but can still be considered arbitrary and results might be sensitive to the chosen range.

### 2.1 Impact of food quality on reproductive performance

In the first assay, we manipulated the diet quality (i.e. yeast availability) of all focal individuals to investigate the effect of dietary stress on reproductive success of males and females in a setup allowing for intra‐sexual competition.

We set up base cultures of 80 individuals of *Ga1* (wildtype) and *Rd* adults in each of the treatments. Stock adults were left to mate and oviposit for 3 days in 60 g of flour according to treatment, afterwards the adults were discarded and the eggs were left to hatch. 31 days after the base cultures had been set up, we collected and sexed pupae and established sex‐separated groups of 40 individuals. 8 days after the last pupae had been sexed, all pupae had emerged and were fully adult. We then set up the mating groups for the experiment.

**FIGURE A1:**
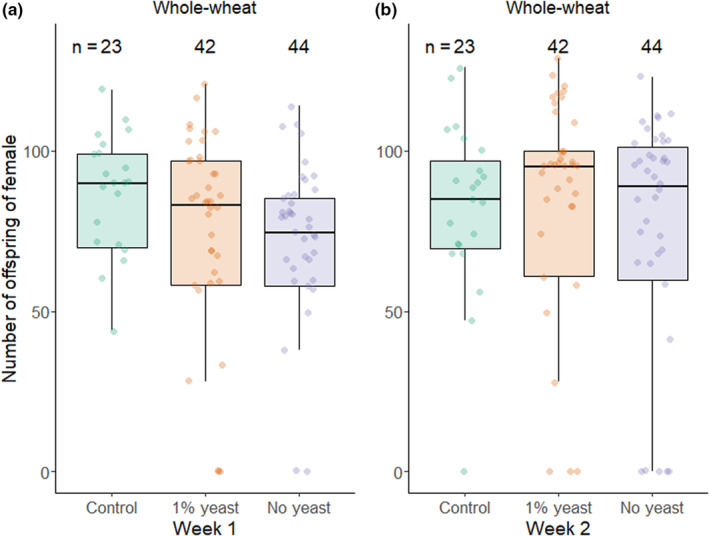
Total wildtype offspring after week one (a) and week two (b) of focal females in whole wheat with varying yeast concentration. Assay with just the focal subjected to treatment.

Groups contained the focal individual (female or male) and a competitor (*Rd*), as well as two mating partners. All mating group beetles were synchronized in age (±3 days) and competitors as well as mating partners were always raised on the control diet. All wildtype mating partners were marked with a dot of *Revell* emaille paint, to differentiate them from the focal. We allowed the treatment groups to mate undisturbed for 3 days in empty arenas (plastic Petri dishes, diameter 3.3 cm). All mating trial arenas were scratched at the bottom to increase traction for the beetles and contained no flour to prevent oviposition. After the mating trial, we froze the males at −30°C for preservation to take phenotypic measurements (see below), while we separated the females to oviposit for 2 weeks in the control environment. In addition, to investigate if females recovered from the treatment when returned to the control diet (i.e. 5% yeast) to lay eggs, we transferred all yeast‐deprived females from the whole‐wheat flour treatment after 1 week to a new egg‐laying vial (Appendix 1: Figure A1). After 2 weeks, females were removed and frozen at −30°C until further processing. Once all offspring reached the adult stage after 50 days, they were frozen and scored for genotype (wildtype or *Rd*; with *Rd* phenotype clearly discernible from the wildtype due to shorter and thicker antennae).

All frozen individuals from the mating groups were weighed to the nearest 0.01 mg on a Sartorius R200D balance (Göttingen, Germany) within 2 months after the main experiment. Body mass was used as a proxy for condition (Rowe & Houle, 1996). The repeatability of body mass, estimated by measuring twice a subset of the samples was high (Intra‐class correlation coefficient, ICC = .937, *p* < .001, *N* = 96).

### 2.2 Impact of food quality on the opportunity for selection

In the second assay, we manipulated the diet quality (i.e. yeast availability) of all individuals (i.e. focals, competitors and mating partners) during development. Hence we mimicked population level dietary manipulation, aiming to measure the opportunity for selection in males and females separately. After the mating trials, all females were given the opportunity to lay eggs on their respective rearing diet (control or yeast deprived). Apart from this, the experimental design followed the procedures described above and was performed in parallel with the first assay.

### 2.3 Statistical analyses

We performed all analyses in R version 4.0.4 (R Core Team, 2018) and used ‘ggplot2’ for creating all figures (Wickham, 2016). In a first series of analyses, we tested for an effect of yeast availability on body mass and reproductive performance. Specifically, we used general linear models (GLMs) with yeast availability defined as the only fixed effect and the body mass (family: Gaussian) or the number of offspring (family: quasi‐Poisson) as response variables. These models were done separately for males and females. For males, we also tested the effect of yeast availability on the genetic mating success (i.e. the number of fertilized females) using GLMs (family: quasi‐Poisson). To test for sex differences, we computed selection coefficients (*s*) as:

$$s=\frac{\left({\bar{W}}_{\text{control}}-{\bar{W}}_{\text{stressed}}\right)}{{\bar{W}}_{\text{control}}}=1-\left(\frac{{\bar{W}}_{\text{stressed}}}{{\bar{W}}_{\text{control}}}\right)$$

With W representing the reproductive success (i.e. offspring number) of control or stressed individuals. Thus, *s* represents a standardized measure for the strength of selection against stressed individuals (Janicke & Chapuis, 2016; Zikovitz & Agrawal, 2013). We used bootstrapping and permutation tests (10,000 permutations) to compare *s* between sexes using the boot package in R (Canty & Ripley, 2019).

In a second series of analyses, we tested the effect of yeast availability on the opportunity for selection, which is defined as the variance in relativized reproductive success (i.e. observed values divided by the group mean) and serves as a proxy for the total net selection (Winkler et al., 2021) in a population (Crow, 1958; Moorad & Wade, 2013; Wade & Shuster, 2005). Similar to the analysis of *s*, we applied bootstrapping to estimate *I* and its 95% confidence intervals (10,000 bootstrap samples) using the boot package in R (Canty & Ripley, 2019). Finally, we decomposed the opportunity for selection of male reproductive success into the variance of the mating success (MS; number of females that produced offspring by the focal male), partner's fecundity (Fec; average number of offspring produced by female partners), paternity share (PS; the proportion of offspring sired per female partner) and each of their covariances. Specifically, we modeled male reproductive success as the product of the genetic mating success, the partner's fecundity, and the paternity share of the focal male (Janicke et al., 2015). To estimate the variance components (with 95% CI) we used bootstrapping (Canty & Ripley, 2019) with 10,000 bootstrap replicates.

## 3 RESULTS

Overall, we observed that development time was considerably longer if yeast was removed from the diet. We did not follow developmental time systematically for all treatments but we collected pupae after 31 and 27 days in the control diet (wheat and whole‐wheat, respectively) and this shifted to 48 and 31 days when yeast was removed from the diet. There were no large differences in development time between 5% and 1% yeast in whole‐wheat flour, with the collection of pupae starting after about 29 days in both treatments. In addition, we tracked the survival of females during 2 weeks after eclosion and observed strongly increased mortality when yeast was constantly removed from the diet. In particular, 19% of females died before the end of the egg‐laying period when no yeast was available in wheat flour, which represents a significantly higher mortality compared to 4% of control females that died at the same time (Pearson's Chi‐squared test with Yates' continuity correction: *df* = 127, *χ* ^2^ = 12.77, *p*‐value < .001). In contrast, in whole‐wheat flour only 0% (control), 0.5% (1% yeast) and 1.1% (no yeast) of females died during the egg laying period (Pearson's Chi‐squared test: *df* = 281, *χ* ^2^ = 1.80, *p*‐value = .407).

### 3.1 Yeast availability influenced body mass only in wheat flour

**FIGURE 1:**
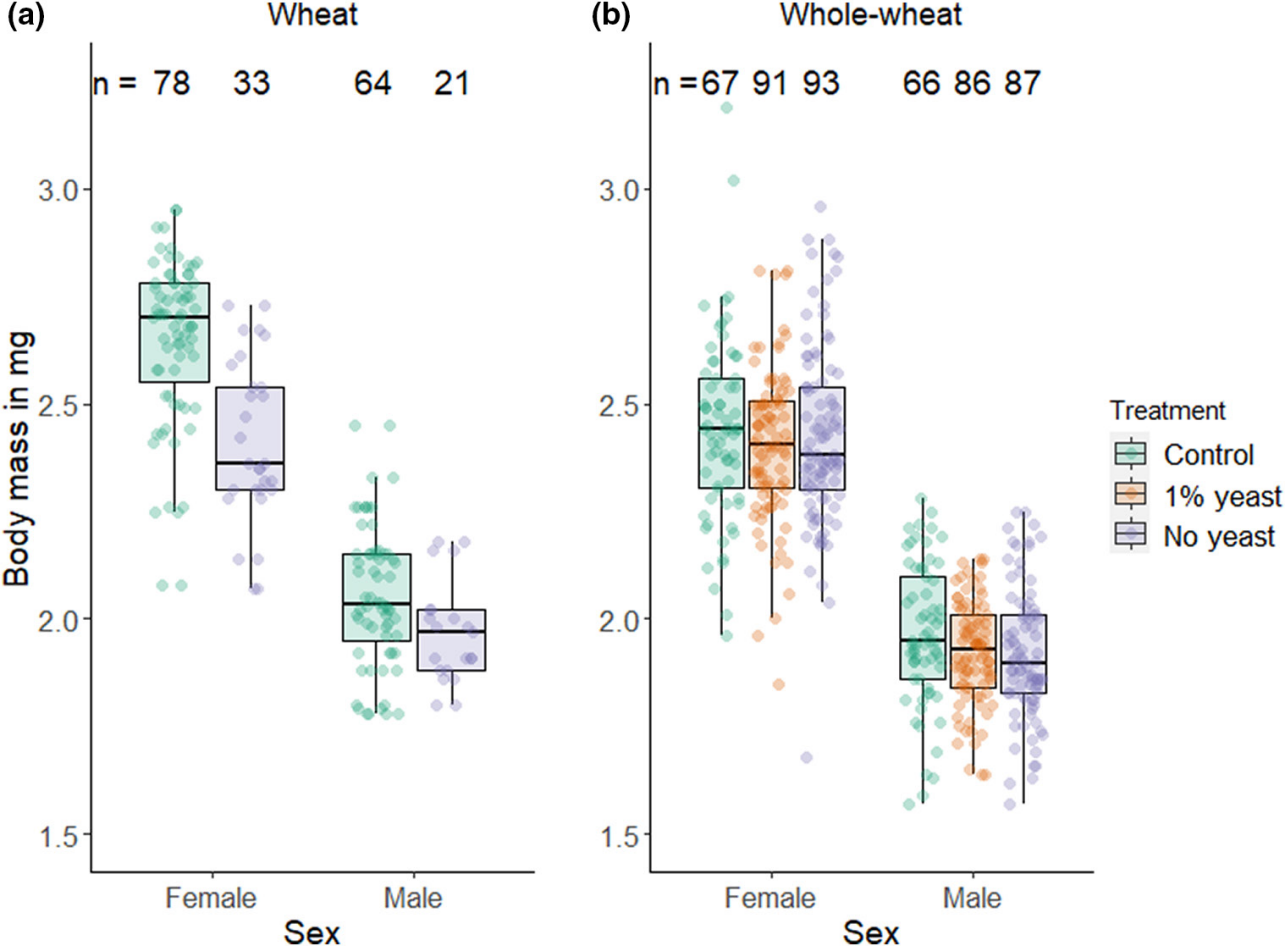
Effect of yeast availability on the body mass of focal males and females observed in (a) wheat flour and in (b) whole‐wheat flour. Boxplots show median, first/third quantiles and whiskers indicating data range excluding outliers. Combined data from both assays of the experiment.

Since our food quality treatment might have affected net energy intake and/or vitamin availability, we measured body mass as a proxy for the condition of focal individuals. For these analyses, we combined data from both assays of the experiment (see Section 2) because the treatment of the focal individual was identical. There was a decrease in body mass in wheat flour when yeast was removed (Figure 1a and Table 1). This effect was significantly larger in females compared to males (GLM on relativized data with sex by treatment interaction: *df* = 181, *t* = 2.05, *p*‐value = .041). Specifically, in females the mean, proportional reduction in body mass was 8.98%, while in males body mass was only reduced by 4.08% compared to the control. By contrast, yeast availability in whole‐wheat did not influence body mass (Figure 1b and Table 1).

**TABLE 1:** Results of general linear models (gaussian) testing the effect of yeast availability on the proportion of offspring produced by the focal individual including ANOVA results for an overall treatment effect and post‐hoc tests.

Flour type	Sex	Treatment effect			Post‐hoc comparisons				
		*df*	*F*‐Value	*p*‐Value	Contrast	Estimate	*SE*	*t*‐Value	Adj. *p*‐Value
Wheat	Male	83	1.98	.034	Control ‐ No yeast	−0.08	.04	−2.15	.034
	Female	98	31.50	<.001	Control ‐ No yeast	−0.24	.04	−5.61	<.001
Whole‐wheat	Male	232	2.34	.100	Control ‐ 1% yeast	−0.04	.02	−1.67	.144
					Control ‐ No yeast	−0.05	.02	−2.08	.116
					1% yeast ‐ No yeast	−0.01	.02	−0.44	.662
	Female	247	0.64	.526	Control ‐ 1% yeast	−0.04	.03	−1.11	.661
					Control ‐ No yeast	−0.01	.03	−0.44	.661
					1% yeast ‐ No yeast	0.02	.03	0.73	.661

*Note*: Combined data from both assays. *p*‐values adjusted for false discovery rate (Benjamini & Hochberg, 1995).

### 3.2 Yeast deprivation impacts reproductive success only in wheat flower

**FIGURE 2:**
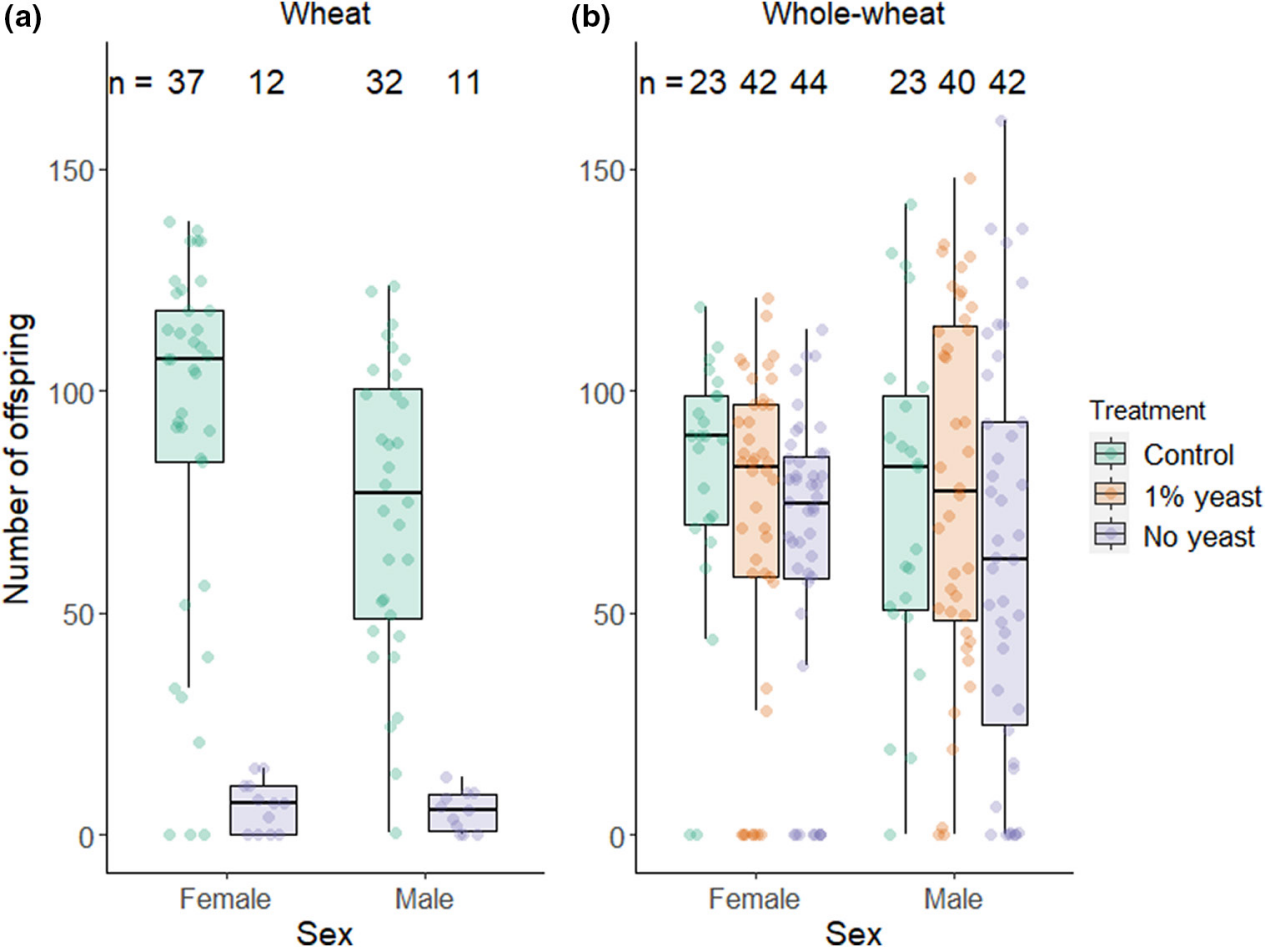
Effect of food availability on (a) number of offspring produced by the focal male or female with or without yeast supplementation in wheat flour and (b) number of offspring produced by the focal male or female with full, 1% or no yeast supplementation in whole‐wheat flour. For males, the total number of offspring was divided by the number of females in the assayed groups (i.e. two) to enable comparison with female values. Only focal animals are subject to treatment (first assay).

In the first assay, we only subjected the focal individual to the treatment while potential competitors and partners were exposed to control food. This enabled us to measure the effect of the treatment on the reproductive success of focal individuals under intra‐sexual competition. There was a significant reduction in the absolute number of offspring produced by females and males in wheat flour if the yeast was removed from their diet (Figure 2a and Table 2). This effect was not significantly different between the sexes (GLM with sex by treatment interaction: *df* = 88, *t* < 0.01, *p*‐value = .998). In contrast, there was no significant reduction in the offspring number of both sexes in whole‐wheat flour if yeast was reduced or removed from the diet (Figure 2b and Table 2).

**TABLE 2:** Results of general linear models (quasi‐Poisson) testing the effect of food availability on the total number of offspring produced by the focal individual including ANOVA results and post‐hoc tests.

Flour type	Sex	Treatment effect			Post‐hoc comparisons				
		*df*	*F*‐Value	*p*‐Value	Contrast	Estimate	SE	*t*‐Value	Adj. *P*‐Value
Wheat	Male	41	81.71	<.001	Control ‐ No yeast	−2.64	.47	−5.66	<.001
	Female	47	88.07	<.001	Control ‐ No yeast	−2.64	.45	−5.85	<.001
Whole‐wheat	Male	102	1.30	.276	Control ‐ 1% yeast	0.04	.15	0.25	.800
					Control ‐ No yeast	−0.17	.16	−1.08	.426
					1% yeast ‐ No yeast	−0.21	.13	−1.55	.372
	Female	106	1.50	.228	Control ‐ 1% yeast	−0.14	.12	−1.10	.413
					Control ‐ No yeast	−0.22	.12	−1.75	.251
					1% yeast ‐ No yeast	−0.08	.11	−0.75	.457

*Note*: Analysis based on experimental assay in which only focal animals have been subjected to treatment. *p*‐Values adjusted for false discovery rate (Benjamini & Hochberg, 1995).

In agreement with the finding that the treatment had a limited influence on the body mass of focal individuals, we also found that body mass did not explain the observed fitness effects of the treatment (Table A1).

### 3.3 No change in egg laying over time

In order to provide a more detailed test on the effect of food quality on female reproductive performance, we examined if egg laying changed over the course of 2 weeks after the mating trials. For this, we transferred females to a fresh vial after the first week of egg laying in the yeast availability experiment to whole‐wheat. A decrease in female reproductive success in the second week of egg laying would indicate that sperm or egg limitation plays a role in female reproductive success. In contrast, an increase in egg laying in week two would indicate that females recovered gradually from negative effects of the previous low‐quality diet. There were no differences in female reproductive success between the first and the second week (Figure A1 and Table A2).

### 3.4 No sex‐specific selection against yeast‐deprived individuals

**FIGURE 3:**
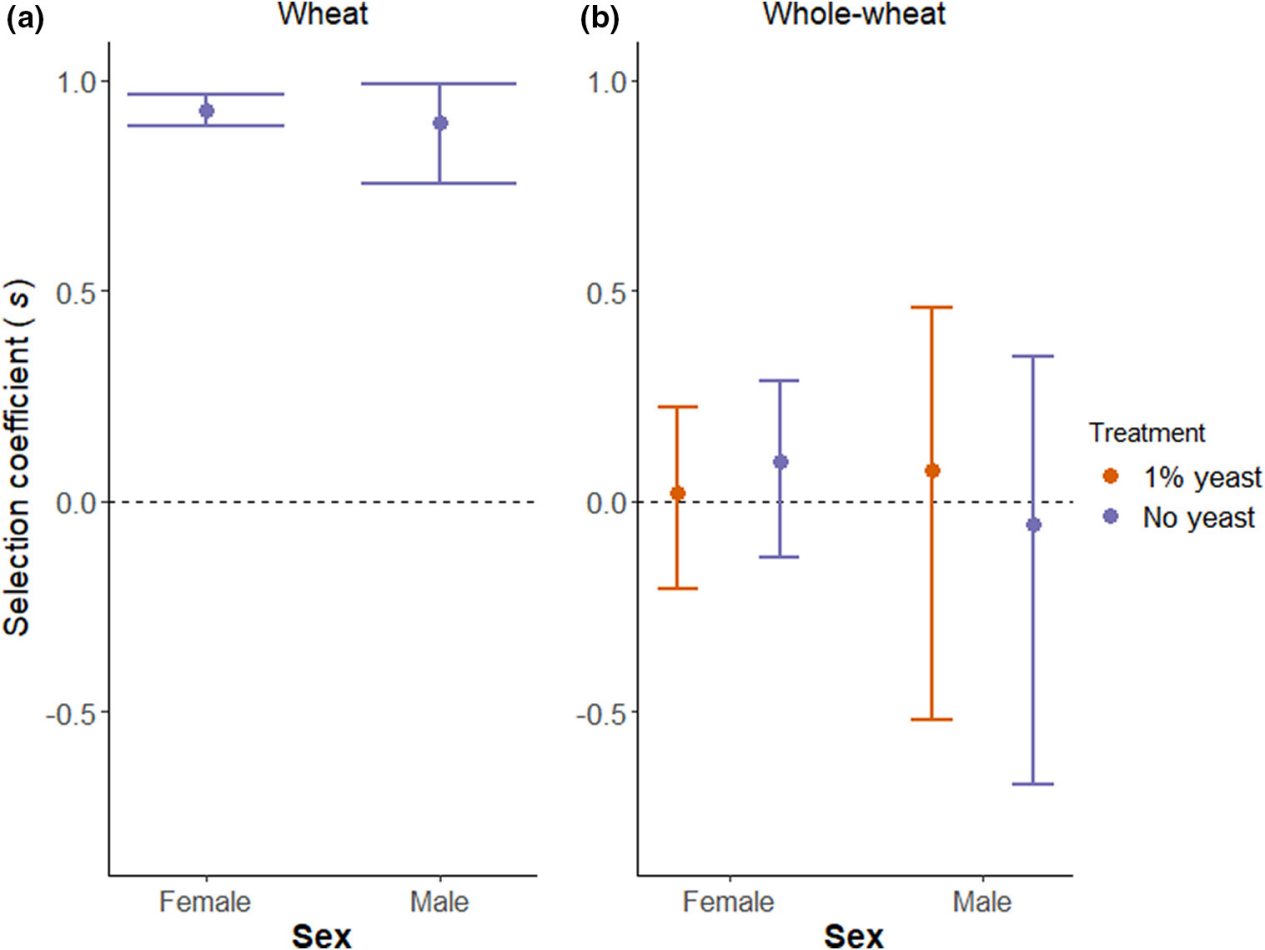
Selection coefficient on the number of offspring produced by the focal male or female for experimental manipulation of yeast availability in wheat flour (a) and in whole‐wheat flour (b). Assay in which only the focal individual was subjected to treatment.

When examining the selection coefficients (a standardized measure for the strength of selection against stressed individuals), there we observed significant selection against yeast‐deprived individuals in wheat flour (Figure 3 and Table 3) but no differences in the strength of selection between males and females (Table 3).

**TABLE 3:** Selection of yeast‐deprived focal individuals and the sex difference in selection coefficients estimated via bootstrapping. Assay in which only the focal individual was subjected to treatment.

Flour type	Treatment	Selection coefficient males (95% CI)	Selection coefficient females (95% CI)	Sex difference (95% CI)
Wheat	No yeast	0.899 (0.759, 0.991)	0.928 (0.889, 0.964)	−0.028 (−0.174, 0.075)
Whole‐wheat	1% yeast	0.071 (−0.544, 0.474)	0.021 (−0.227, 0.218)	0.051 (−0.581, 0.517)
	No yeast	−0.058 (−0.689, 0.349)	0.093 (−0.135, 0.278)	−0.151 (−0.836, 0.311)

### 3.5 Lower mating success in yeast‐deprived males

**FIGURE A2:**
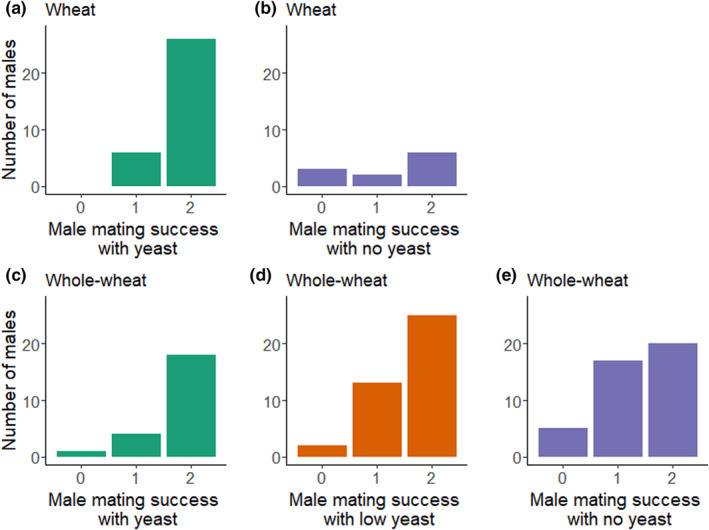
Histograms of genetic mating success of focal males in wheat (a, b) and whole‐wheat flour (c–e) with yeast supplementation (a, c), without yeast supplementation (b, e) or low yeast supplementation (d). Only focal males subject to treatment.

For males, we were able to estimate the genetic mating success (number of fertilized females) as a fitness component contributing to the measured reproductive success. In the assay in which only the focal was treated, yeast deprivation had a negative effect on the genetic mating success of males in wheat flour (Figure A2a,b and Table 4). Nevertheless, there was no difference in the genetic mating success of males in whole‐wheat (Figure A2c–e and Table 4).

**TABLE 4:** Results of general linear models (quasi‐Poisson) testing for an effect of food quality on the genetic mating success of focal males including ANOVA results and post‐hoc tests.

Flour type	Treatment effect			Post‐hoc comparisons				
	*df*	*f*	*p*‐Value	Contrast	Estimate	*SE*	*t* value	Adj. *p*‐Value
Wheat	41	6.79	.013	Control ‐ No yeast	−0.35	.14	−2.52	.016
Whole‐wheat	102	2.90	.059	Control ‐ 1% yeast	−0.10	.10	−0.95	.342
				Control ‐ No yeast	−0.25	.11	−2.34	.064
				1% yeast ‐ No yeast	−0.15	.09	−1.58	.174

*Note*: Assay in which only the focal subjected to treatment. *p*‐values adjusted for false discovery rate (Benjamini & Hochberg, 1995).

### 3.6 Sex‐specific effects of diet on the opportunity for selection

**FIGURE 4:**
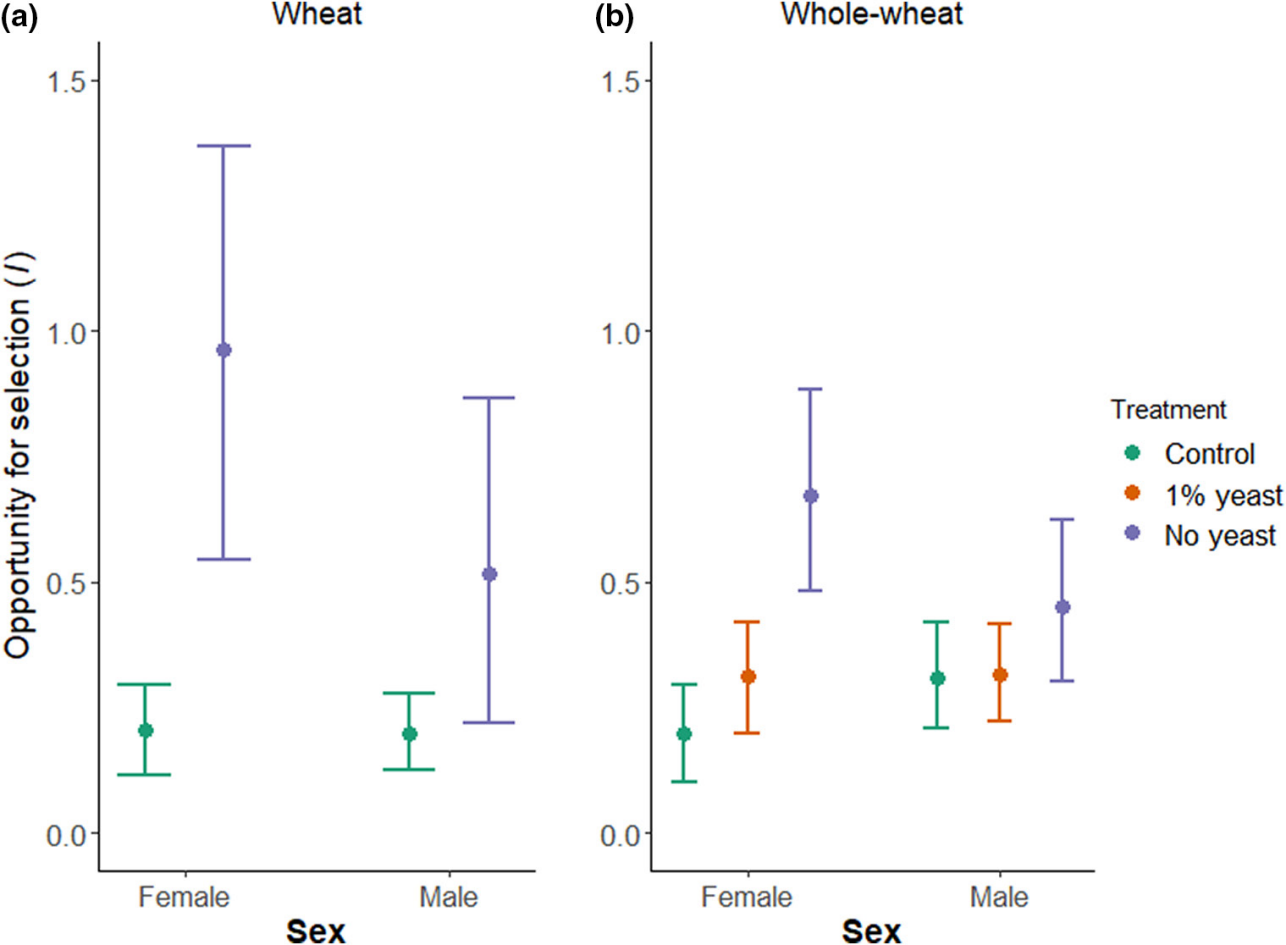
Effect of yeast availability on the opportunity for selection (*I*) estimated as the variance of relativized number of offspring by the focal males and females in wheat flour (a) and in whole‐wheat flour (b). Assay for measuring the opportunity for selection, with all individuals in the group subject to treatment. Bars show means and 95% CI of *I* obtained from bootstrapping.

**FIGURE A3:**
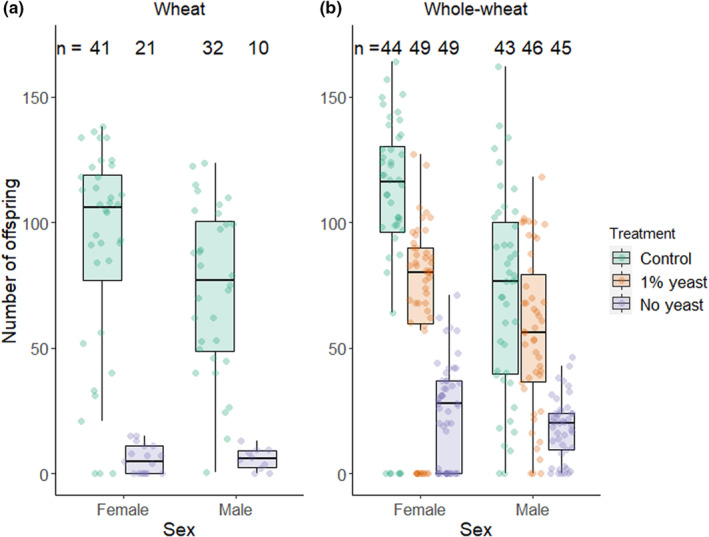
Effect of food availability on (a) number of offspring produced by the focal male or female with or without yeast supplementation in wheat flour and (b) number of offspring produced by the focal male or female with full, 1% or no yeast supplementation in whole‐wheat flour. For males, the total number of offspring was divided by the number of females in the assayed groups (i.e. two) to enable comparison with female values. Assay for measuring the opportunity for selection, with all animals subject to treatment.

One main objective of our study was to test if a low‐quality diet (i.e. no yeast supplementation) affected the opportunity for selection (*I*) in *T. castaneum* in a sex‐specific manner. Therefore, we manipulated the diet quality of all individuals (i.e. focal, potential mating partners and potential competitors) of the mating groups in a second experimental assay to mimic selection in a population under dietary stress. We measured *I* as the variance in relative reproductive success of the focal. We found that in this assay, there was a significant effect of the diet treatment on reproductive success of both sexes in wheat as well as whole‐wheat flour (Figure A3 and Table A3). Importantly, we found that *I*_Females_ was higher when yeast was removed from the diet (Figure 4a and Table 5). We also detected an increase in *I*_Males_ but this effect was less pronounced and statistically non‐significant (Figure 4a and Table 5). Likewise, in whole‐wheat flour the absence of yeast increased *I*_Females_ compared to the control, but *I*_Males_ only increased slightly (Figure 4b and Table 5). The reduction of yeast to 1% did not increase the opportunity for selection in either of the sexes compared to the control. Finally, there was only a non‐significant trend towards larger *I*_Females_ compared to *I*_Males_ in wheat and whole‐wheat when yeast was removed from the diet (Table A4).

**TABLE 5:** Opportunity for selection (*I)* estimated as the variance of relativized number of offspring estimated via bootstrapping.

Flour type	Sex	Treatment	*I* (95%CI)
Wheat	Male	Control	0.197 (0.124, 0.277)
		No yeast	0.517 (0.215, 0.869)
	Female	Control	0.207 (0.113, 0.296)
		No yeast	0.961 (0.542, 1.353)
Whole‐wheat	Male	Control	0.308 (0.208, 0.421)
		1% yeast	0.317 (0.223, 0.418)
		No yeast	0.451 (0.298, 0.623)
	Female	Control	0.200 (0.103, 0.296)
		1% yeast	0.310 (0.193, 0.417)
		No yeast	0.671 (0.481, 0.883)

*Note*: Assay for measuring the opportunity for selection, with all individuals subject to treatment.

### 3.7 Variance decomposition for focal males

**FIGURE 5:**
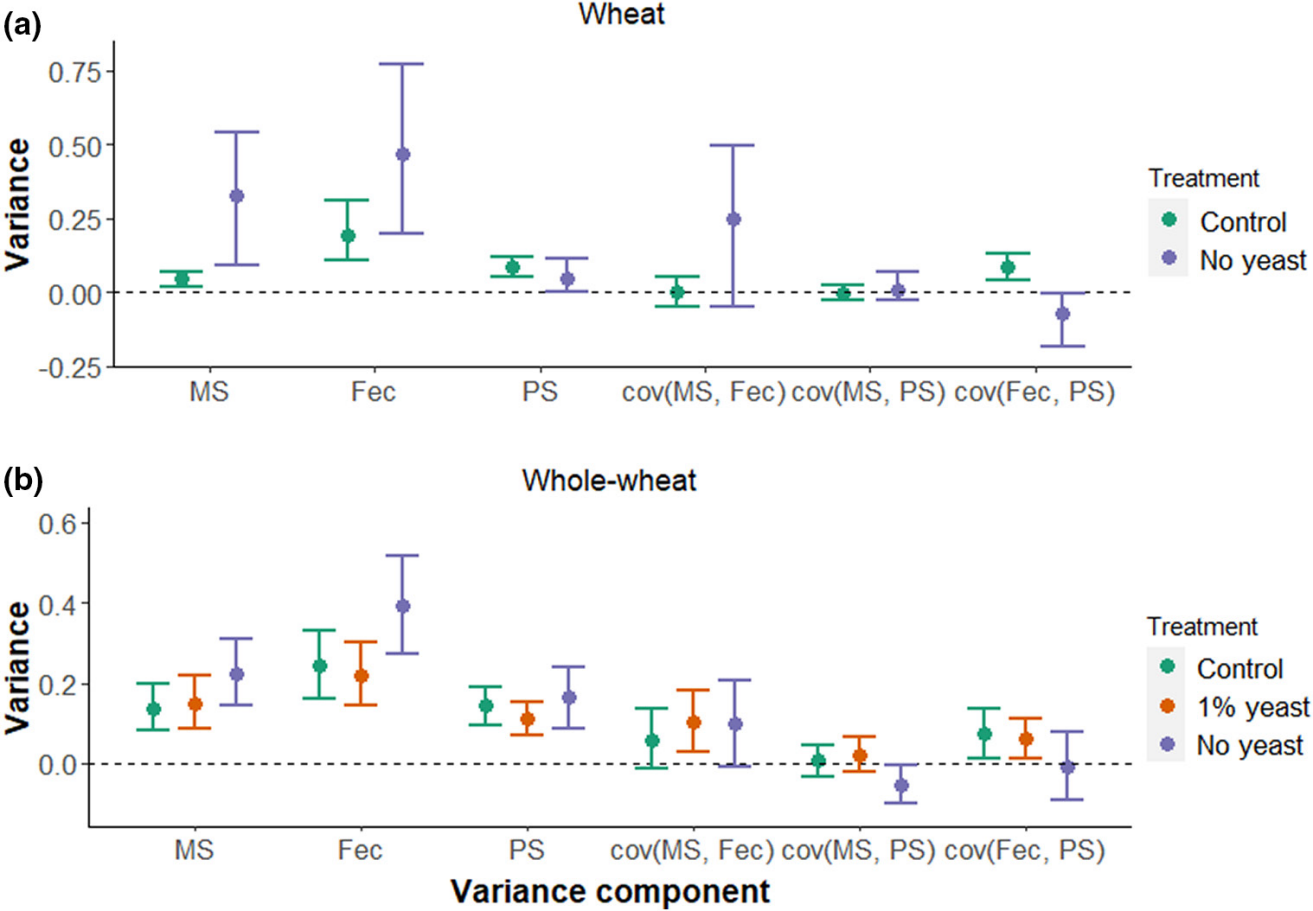
Effects of yeast availability on the variance of relativized male mating success (MS), partner's fecundity (Fec), paternity share (PS) and the respective covariances in wheat flour (a) and in whole‐wheat flour (b). Analysis based on experimental assay in which all individuals have been subjected to the food treatment. Means of bootstrapped variances and 95% CIs are shown.

To better understand the observed effects of food quality on the opportunity for selection in males, we partitioned male reproductive success into three main fitness components: genetic mating success, paternity share and partner's fecundity. We found that the variance in mating success was higher in the no‐yeast treatment in wheat‐flour compared to the control (Figure 5a and Table A5), though the 95% CI overlaps slightly. There was also a trend for a larger variance in partner's fecundity, while there was no difference in the variance in paternity share. In whole‐wheat, the effects of yeast availability on the variances in mating success and partner's fecundity were smaller but showed the same direction as observed in wheat flour (Figure 5b and Table A5).

### 3.8 Lower body mass and poor fitness of *Rd* males

**FIGURE A4:**
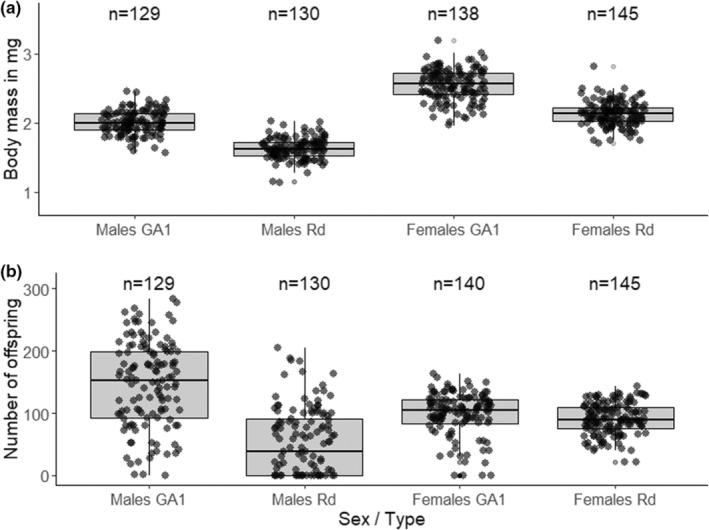
Body mass (a) and number of offspring (b) of wildtype (Ga1) focals and *Rd* competitors. Shown data only includes control treatment. Assay 1 & 2 combined.

Finally, we aimed to evaluate the competitiveness of the used *Rd* mutant strain in both assays of the experiment by comparing the body mass and reproductive success of *Rd* competitors with the wildtype focal. Overall, we found *Rd* of both sexes to have lower body mass compared to wildtype (Figure A4 and Tables A6 and A7). Furthermore, *Rd* males had significantly fewer offspring compared to wildtype males (65% less; Figure A4 and Table A6). In contrast, there was no difference in offspring numbers of *Rd* females compared to wildtype females (Figure A4 and Table A7).

## 4 DISCUSSION

This study demonstrates that low food quality impairs the reproductive performance of both sexes and affected the opportunity for selection in the red flour beetle *Tribolium castaneum*. More specifically, our data reveal four main findings: (i) body mass, female survival, male mating success and offspring production were negatively impacted by removing yeast from a wheat diet, (ii) the magnitude of the effect of poor diet on offspring production was similar for males and females, (iii) the negative effects of yeast deprivation observed on wheat diet were buffered or undetectable on a whole‐wheat flour diet, and (iv) a poor diet quality inflated the opportunity for selection primarily for females.

### 4.1 Negative impact of yeast deprivation only in wheat flour

Yeast deprivation reduced body mass significantly on a wheat but not on a whole‐wheat diet. This effect was greater in females compared to males suggesting that yeast had a stronger effect on female condition. The absence of an effect of yeast deprivation in whole‐wheat indicates that whole‐wheat mitigates the negative impact of yeast reduction in both sexes. Interestingly, the reduction in body mass did not explain the observed effect of diet on reproductive success in both sexes (see below), which is in line with a previous study reporting no effect of lowered body mass on fitness in *T. castaneum* cultured on a low‐quality diet using a non‐nutritional filler (Plesnar‐Bielak et al., 2017). We note that the present study might underestimate the influence of yeast deprivation on body mass because rearing density was presumably not balanced across treatments. In particular, we expect that density during development was lower for the yeast‐deprived focal individuals due to a reduced hatching success and/or survival of larvae and pupae. This could have led to an underestimation of the effect of the yeast deprivation, as high density might negatively impact body mass in insects (Morimoto et al., 2016; Than et al., 2020).

Furthermore, we observed that yeast availability affected development time and had a strong impact on the mortality of focal females during the experiment, but only in wheat and not in whole‐wheat flour. However, we only estimated mortality for females over a two‐week period, which is relatively short given an average reproductive lifespan of 0.5–1 year (Fedina & Lewis, 2008) and a total lifespan of up to 4 years (Pointer et al., 2021). In addition, our findings on development time are rather exploratory since we only measured the time needed for cultures to yield a sufficient number of pupae for sexing rather than tracking individual development. Nevertheless, previous studies on effects of yeast‐deprived diet in *Tribolium* also reported a significant increase in development time (Sokoloff et al., 1966). These findings on development, survival and body mass suggest that our experimental manipulation of yeast availability affected the pool of resources (i.e. condition) of focal males and females in the wheat diet treatment but not necessarily in the whole‐wheat diet. However, condition itself is difficult to measure and certainly not fully captured by body mass (Barnett et al., 2015; Wilder et al., 2016). Moreover, diet manipulation may have sex‐specific effects that are independent of condition. For these reasons, we stress that our findings on the effect of diet on reproductive performance should not be interpreted solely in the context of condition‐dependence. The main focus of our study was to explore the causal link between dietary stress and reproductive performance, which can, but does not have to be mediated by the effects of diet on condition.

Most importantly, we found a negative impact of yeast deprivation on male and female reproductive success in wheat flour. For males, this is in line with the previous finding that investment into spermatogenesis and testes mass was reduced under yeast deprivation in wheat flour (Godwin et al., 2017). In contrast, we did not detect statistically significant effects of yeast availability in whole‐wheat flour indicating that the higher nutritional quality of whole‐wheat flour (Kumar et al., 2011; Likes et al., 2007) buffered the negative effects of removing baker's yeast from the diet of *T. castaneum*. Opposite to our prediction of higher condition‐dependence of male reproductive success as a consequence of male‐biased sexual selection, our results suggest that condition had a similar effect on reproductive success in both sexes. Remarkably, male genetic mating success (i.e., number of successfully inseminated females) was lower in yeast‐deprived individuals that grew on wheat flour. Therefore, the observed effect of yeast availability on male reproductive success might, at least partially, be driven by a lowered competitiveness at pre‐copulatory episodes of sexual selection.

In the context of what is known from other model systems, our findings support the idea that sex‐specific effects of dietary stress on reproductive success can vary across species. In the fruit fly *Drosophila melanogaster*, low food quality provided during development had a stronger negative effect on reproductive success in males compared to females (Zikovitz & Agrawal, 2013). By contrast, a study on the freshwater snail *Physa acuta* suggests that dietary stress impairs primarily female reproductive success (Janicke & Chapuis, 2016), though this finding might be a consequence of condition‐dependent sex allocation, which is restricted to simultaneous hermaphrodites. The observed absence of a sex‐specific‐effect of food quality on reproductive success in *T. castaneum* may have at least three reasons. First, the alleged sex difference in the strength of sexual selection might actually be too small to generate sex‐specific stress responses. Several lines of empirical work suggest that sexual selection is stronger in males and operates primarily along post‐copulatory episodes (Fedina & Lewis, 2008). However, a formal comparison of the standardized metrics (e.g. the Bateman gradient or Jones index [Jones, 2009]) for the strength of sexual selection between males and females is still lacking for *T. castaneum*. Second, yeast availability may not only affect the individual's condition in males and females but may also contain compounds/elements that are more critical for egg production rather than for sperm production or any other sexually selected trait of males. Hence, female reproduction might be more sensitive to yeast deprivation despite higher condition‐dependence of male reproductive success. Nevertheless, a previous study manipulating yeast availability in wheat flour in *T. castaneum* males found an effect on spermatogenesis and testes investment, suggesting a marked effect on male reproductive performance (Godwin et al., 2017). Third, the absence of sex‐specific stress response may also reflect the methodological limitations of our experimental setup. Specifically, body mass of both sexes and male reproductive success of the *Rd* mutant strain (which served as competitors during mating trials) was considerably lower compared to wildtype individuals, which has already been found in previous studies (Godwin et al., 2018; Sbilordo & Martin, 2014). Hence, competition for mates and/or their gametes might have been low for males, which may translate into an underestimation of condition‐dependence of male reproductive performance. In addition, we only measured reproductive success over an egg‐laying period of 2 weeks, therefore we did not capture possible effects of diet quality on reproductive senescence. Further work on the effect of food availability is clearly needed to fully understand the effect of dietary stress on male and female reproductive success in *Tribolium* beetles. This may include experimental setups that apply alternative methods to manipulate conditions (e.g. food quantity rather than quality) and studies testing explicitly the effect of pre‐ and post‐copulatory competition on diet‐induced stress responses in males and females. Moreover, the apparent inter‐specific variation in sex‐specific stress responses and its evolutionary link to differences in the strength of sexual selection on males and/or females across species constitutes a promising avenue to follow using comparative approaches.

### 4.2 Yeast availability inflates the opportunity for selection

The main objective of the second mating assay (i.e. all individuals where subjected to the treatment) was to test for an effect of food quality on the opportunity of selection (*I*) and whether this effect differs between males and females. As predicted, a reduction in food quality increased *I* in both sexes though this effect was statistically significant only in females under both wheat flour and whole‐wheat flour. This result is in line with the prediction that environmental stress may unmask cryptic genetic variation and therefore inflate *I* (Paaby & Rockman, 2014). Similar to our findings, food stress has been found to increase *I* of both sex functions in the freshwater snail *P. acuta* (Janicke et al., 2022). Interestingly, our variance decomposition of male reproductive success suggests that the increase of *I* in yeast‐deprived populations can partly be attributed to a higher variance in mating success (also called the ‘opportunity for sexual selection’). This suggests that pre‐copulatory sexual selection in terms of male–male competition is higher in yeast‐deprived populations of *T. castaneum*.

Sexual selection theory predicts that stronger sexual selection on males manifests in a higher *I*_Males_ (Bateman, 1948), which is well supported across a broad range of animal taxa (Janicke et al., 2016; Winkler et al., 2021). Our data do not support this hypothesis as we did not detect consistent sex differences in *I* across the tested food treatments. On the assumption that phenotypic variance in reproductive success translates into genetic variation in fitness, this finding may have implications for the adaptation to stressful food conditions. This is because populations are expected to purge deleterious alleles at a low demographic cost only if net selection (measured in terms of genetic variance in fitness but often approximated by estimating *I*) is stronger on males (Whitlock & Agrawal, 2009). Our data do not indicate stronger net selection on males and hence, under the tested experimental conditions, sexual selection would not be expected to purge deleterious alleles at low demographic costs. However, as mentioned before, male–male competition was presumably limited in our experimental setup, which may have restricted *I*_Males_ to be low and the sex difference in net selection might be more male‐biased under conditions allowing for more intense male–male competition.

## 5 CONCLUSION

Overall, our study demonstrates that yeast restriction can impose major fitness costs on male and female *T. castaneum* beetles, which can be partially buffered by higher nutritional quality of whole‐wheat compared to wheat flour. Moreover, a lowered diet quality leads to an increased opportunity for selection especially in females, suggesting high demographic costs for the adaptation to environments of poor diet quality.

## AUTHOR CONTRIBUTIONS

**Lennart Winkler:** Conceptualization (equal); data curation (lead); formal analysis (lead); investigation (lead); methodology (equal); resources (equal); software (lead); visualization (lead); writing – original draft (lead); writing – review and editing (equal). **Tim Janicke:** Conceptualization (equal); formal analysis (supporting); funding acquisition (lead); methodology (equal); project administration (lead); resources (equal); supervision (lead); visualization (supporting); writing – original draft (supporting); writing – review and editing (equal).

## FUNDING INFORMATION

This study was funded by the *Deutsche Forschungsgemeinschaft* (DFG) under the grant reference number ‘JA 2653/2–1’. The DFG had no role in the design of the study and collection, analysis, and interpretation of data and in writing the manuscript.

## CONFLICT OF INTEREST

The authors declare that they have no competing interests.

## Data Availability

All data analyzed in this study are uploaded to the Dryad data repository (https://doi.org/10.5061/dryad.05qfttf6h; Winkler & Janicke, 2022).

## APPENDIX 1

### Influence of body mass on the effect of treatment on fitness

**TABLE A1:** ANOVA of general linear models (GLMs) of quasi‐Poisson family modeling the influence of yeast availability in wheat and whole‐wheat flour on offspring number of focal when including body mass.

Design	Flour type	Sex	Coefficient	*df*	*F*‐Value	*p*‐Value
Only focal subject to treatment	Wheat flour	Male	Treatment	39	76.57	<.001
			Focal body mass [mg]		0.01	.917
			Treatment × body mass		0.36	.552
		Female	Treatment	44	63.78	<.001
			Focal body mass [mg]		0.19	.667
			Treatment × body mass		<0.01	.984
All animals subject to treatment	Wheat flour	Male	Treatment	38	68.20	<.001
			Focal body mass [mg]		0.01	.917
			Treatment × body mass		0.32	.574
		Female	Treatment	48	93.46	<.001
			Focal body mass [mg]		0.22	.638
			Treatment × body mass		<0.01	.959
Only focal subject to treatment	Whole‐wheat flour	Male	Treatment	98	1.19	.310
			Focal body mass [mg]		0.71	.403
			Treatment x body mass		1.04	.355
		Female	Treatment	103	1.07	.347
			Focal body mass [mg]		2.56	.112
			Treatment × body mass		4.92	.009
All animals subject to treatment	Whole‐wheat flour	Male	Treatment	125	44.90	<.001
			Focal body mass [mg]		1.84	.177
			Treatment × body mass		0.42	.660
		Female	Treatment	135	62.28	<.001
			Focal body mass [mg]		1.65	.201
			Treatment × body mass		2.88	.060

### Egg laying success over time

**TABLE A2:** ANOVA of general linear model of quasi‐Poisson family comparing the total number of offspring sired by the focal female in week one and two of the whole‐wheat experiment separately for each treatment.

Flour type	Contrast	*df*	Estimate	*SE*	*F*‐Value	*p*‐Value
Control	Week 1 vs. 2	44	<−0.01	.12	<0.01	.966
Low yeast	Week 1 vs. 2	82	0.14	.11	1.46	.230
No yeast	Week 1 vs. 2	86	0.15	.12	1.57	.214

*Note*: Assay with just the focal subjected to treatment.

### Additional analyses for the assay for measuring the opportunity for selection

**TABLE A3:** Results of general linear models (quasi‐Poisson) modeling the effect of food quality on the total number of offspring produced by the focal including ANOVA results and post‐hoc tests.

Flour type	Sex	Treatment effect			Post‐hoc comparisons				
		*df*	*F*‐Value	*p*‐Value	Contrast	Estimate	*SE*	*t*‐value	Adj. *p*‐Value
Wheat	Male	40	72.84	<.001	Control ‐ No yeast	−2.54	.47	−5.44	<.001
	Female	51	124.35	<.001	Control ‐ No yeast	−2.77	.40	−6.92	<.001
Whole‐wheat	Male	131	48.88	<.001	Control ‐ 1% yeast	−0.24	.11	−2.25	.026
					Control ‐ No yeast	−1.37	.16	−8.64	<.001
					1% yeast ‐ No yeast	−1.13	.16	−6.98	<.001
	Female	138	62.43	<.001	Control ‐ 1% yeast	−0.43	.10	−4.21	<.001
					Control ‐ No yeast	−1.43	.14	−9.98	<.001
					1% yeast ‐ No yeast	−1.00	.15	−6.71	<.001

*Note*: Assay for measuring the opportunity for selection, with all animals subject to treatment. *p*‐values adjusted for false discovery rate.

### Sex bias in the opportunity for selection

**TABLE A4:** Sex bias in variance on number of offspring by the focal estimated via bootstrapping. Experiment with all animals subject to treatment.

Flour type	Treatment	Sex bias	Lower 95% CI	Upper 95% CI	*p*‐Value
Wheat	Control	−0.010	−0.125	0.112	.887
	No yeast	−0.443	−0.959	0.098	.057
Whole‐wheat	Control	0.108	−0.034	0.255	.649
	1% yeast	0.007	−0.137	0.159	.306
	No yeast	−0.219	−0.479	0.035	.094

*Note*: Positive sex bias values indicate male bias. *p*‐values estimated via permutation test.

### Variance decomposition for focal males

**TABLE A5:** Variance of standardized relative reproductive success estimated via bootstrapping.

Flour type	Variance component	Control estimate (95%CI)	Low yeast (95%CI)	No yeast (95%CI)
Wheat	MS	0.046 (0.018, 0.071)	NA	0.327 (0.051, 0.504)
	Fec	0.196 (0.109, 0.309)	NA	0.467 (0.193, 0.763)
	PS	0.087 (0.056, 0.121)	NA	0.045 (<0.001, 0.111)
	cov(MS, Fec)	0.005 (−0.047, 0.053)	NA	0.246 (−0.054, 0.501)
	cov(MS, PS)	−0.004 (−0.028, 0.023)	NA	0.011 (−0.026, 0.072)
	cov(Fec, PS)	0.085 (0.040, 0.133)	NA	−0.073 (−0.189, <−0.001)
Whole‐wheat	MS	0.134 (0.082, 0.197)	0.148 (0.086, 0.213)	0.225 (0.143, 0.308)
	Fec	0.241 (0.162, 0.328)	0.220 (0.146, 0.304)	0.391 (0.271, 0.512)
	PS	0.145 (0.095, 0.192)	0.112 (0.071, 0.154)	0.165 (0.088, 0.240)
	cov(MS, Fec)	0.058 (−0.013, 0.137)	0.102 (0.028, 0.182)	0.100 (−0.006, 0.210)
	cov(MS, PS)	0.006 (−0.034, 0.047)	0.020 (−0.021, 0.068)	−0.053 (−0.102, −0.004)
	cov(Fec, PS)	0.075 (0.014, 0.136)	0.061 (0.012, 0.111)	−0.001 (−0.092, 0.079)

*Note*: Assay for measuring the opportunity for selection, with all animals subject to treatment.

### *Rd* mass and competitiveness

**TABLE A6:** ANOVA of general linear model of gaussian family modeling the differences in body mass between competitor *Rd* and wildtype individuals.

Sex	Mean wildtype	Mean *Rd*	*df*	*F*‐Value	*p*‐Value
Male	2.01	1.62	127	360.25	<.001
Female	2.55	2.13	136	301.75	<.001

*Note*: Data only including control treatment. Assay 1 & 2 combined.

**TABLE A7:** ANOVA of general linear model of quasi‐Poisson family modeling the differences in offspring number between competitor *Rd* and wildtype individuals.

Sex	Mean wildtype	Mean *Rd*	*df*	*F*‐Value	*p*‐Value
Male	146.33	51.75	128	143.51	<.001
Female	92.59	91.08	138	0.14	.711

*Note*: Data only including control treatment. Assay 1 & 2 combined.

## References

[ece39533-bib-0001] Abdel‐Tawwab, M. , Abdel‐Rahman, A. M. , & Ismael, N. E. M. (2008). Evaluation of commercial live bakers’ yeast, *Saccharomyces cerevisiae* as a growth and immunity promoter for fry Nile tilapia, *Oreochromis niloticus* (L.) challenged in situ with *Aeromonas hydrophila* . Aquaculture, 280(1–4), 185–189. 10.1016/j.aquaculture.2008.03.055

[ece39533-bib-0002] Barnett, C. A. , Suzuki, T. N. , Sakaluk, S. K. , & Thompson, C. F. (2015). Mass‐based condition measures and their relationship with fitness: In what condition is condition? Journal of Zoology, 296(1), 1–5. 10.1111/jzo.12213 26019406 PMC4442632

[ece39533-bib-0003] Bateman, A. J. (1948). Intra‐sexual selection in *drosophila* . Heredity, 2, 349–368. 10.1038/hdy.1948.21 18103134

[ece39533-bib-0004] Benjamini, Y. , & Hochberg, Y. (1995). Controlling the false discovery rate: A practical and powerful approach to multiple testing. Journal of the Royal Statistical Society: Series B: Methodological, 57(1), 289–300. 10.2307/2346101

[ece39533-bib-0005] Berger, D. , Martinossi‐Allibert, I. , Grieshop, K. , Lind, M. I. , Maklakov, A. A. , & Arnqvist, G. (2016). Intralocus sexual conflict and the tragedy of the commons in seed beetles. The American Naturalist, 188(4), E98–E112. 10.1086/687963 27622882

[ece39533-bib-0006] Bonduriansky, R. , Maklakov, A. , Zajitschek, F. , & Brooks, R. (2008). Sexual selection, sexual conflict and the evolution of ageing and life span. Functional Ecology, 22(3), 443–453. 10.1111/j.1365-2435.2008.01417.x

[ece39533-bib-0007] Brankatschk, M. , Dunst, S. , Nemetschke, L. , & Eaton, S. (2014). Delivery of circulating lipoproteins to specific neurons in the *drosophila* brain regulates systemic insulin signaling. eLife, 3, 1–19. 10.7554/eLife.02862 PMC4210815 25275323

[ece39533-bib-0008] Brankatschk, M. , Gutmann, T. , Knittelfelder, O. , Palladini, A. , Prince, E. , Grzybek, M. , Brankatschk, B. , Shevchenko, A. , Coskun, Ü. , & Eaton, S. A. (2018). A temperature‐dependent switch in feeding preference improves *drosophila* development and survival in the cold. Developmental Cell, 46(6), 781–793.e4. 10.1016/j.devcel.2018.05.028 30253170

[ece39533-bib-0009] Brooker, R. M. , Jones, G. P. , & Munday, P. L. (2013). Prey selectivity affects reproductive success of a corallivorous reef fish. Oecologia, 172(2), 409–416. 10.1007/s00442-012-2521-7 23124333

[ece39533-bib-0010] Cally, J. G. , Stuart‐Fox, D. , & Holman, L. (2019). Meta‐analytic evidence that sexual selection improves population fitness. Nature Communications, 10, 2017. 10.1038/s41467-019-10074-7 PMC6494874 31043615

[ece39533-bib-0011] Canty, A. , & Ripley, B. (2019). Boot: Bootstrap R (S‐plus) functions . R package version, 1, 3‐20.

[ece39533-bib-0012] Chapman, R. N. (1924). Nutritional studies on the confused flour beetle *Tribolium confusum duval* . Journal of General Physiology, 451, 565–585. 10.1085/jgp.6.5.565 PMC2140673 19872096

[ece39533-bib-0013] Crow, J. F. (1958). Some possibilities for measuring selection intensities in man. Human Biology, 30(1), 1–13. 13513111

[ece39533-bib-0014] Duxbury, E. M. L. , & Chapman, T. (2020). Sex‐specific responses of life span and fitness to variation in developmental versus adult diets in *Drosophila melanogaster* . The Journals of Gerontology. Series A, Biological Sciences and Medical Sciences, 75(8), 1431–1438. 10.1093/GERONA/GLZ175 31362304 PMC7357588

[ece39533-bib-0015] Eldrigde, J. L. , & Krapu, G. L. (1988). The influence of diet quality on clutch size and laying pattern in mallards. The Auk, 105(1), 102–110. 10.1093/auk/105.1.102

[ece39533-bib-0016] Fedina, T. Y. , & Lewis, S. M. (2008). An integrative view of sexual selection in *Tribolium flour* beetles. Biological Reviews, 83, 151–171. 10.1111/j.1469-185X.2008.00037.x 18429767

[ece39533-bib-0017] García‐González, R. , Aldezabal, A. , Laskurain, N. A. , Margalida, A. , & Novoa, C. (2016). Influence of snowmelt timing on the diet quality of pyrenean rock ptarmigan (*Lagopus muta pyrenaica*): Implications for reproductive success. PLoS One, 11(2), e0148632. 10.1371/journal.pone.0148632 26849356 PMC4746074

[ece39533-bib-0018] Geister, T. L. , Lorenz, M. W. , Hoffmann, K. H. , & Fischer, K. (2008). Adult nutrition and butterfly fitness: Effects of diet quality on reproductive output, egg composition, and egg hatching success. Frontiers in Zoology, 5, 1–13. 10.1186/1742-9994-5-10 18616795 PMC2481257

[ece39533-bib-0019] Godwin, J. L. , Spurgin, L. G. , Michalczyk, Ł. , Martin, O. Y. , Lumley, A. J. , Chapman, T. , & Gage, M. J. (2018). Lineages evolved under stronger sexual selection show superior ability to invade conspecific competitor populations. Evolution Letters, 2(5), 511–523. 10.1002/evl3.80 30283698 PMC6145403

[ece39533-bib-0020] Godwin, J. L. , Vasudeva, R. , Michalczyk, Ł. , Martin, O. Y. , Lumley, A. J. , Chapman, T. , & Gage, M. J. (2017). Experimental evolution reveals that sperm competition intensity selects for longer, more costly sperm. Evolution Letters, 1(2), 102–113. 10.1002/evl3.13 30283643 PMC6089504

[ece39533-bib-0021] Good, N. (1933). Biology of the flour beetles, *Tribolium confusum Duv*. And *T. ferrugineum fab* . Journal of Agricultural Research, 46(4), 327–334.

[ece39533-bib-0022] Guo, R. , & Reinhardt, K. (2020). Dietary polyunsaturated fatty acids affect volume and metabolism of *Drosophila melanogaster* sperm. Journal of Evolutionary Biology, 33(4), 544–550. 10.1111/jeb.13591 31961473

[ece39533-bib-0023] James, C. M. , Dias, P. , & Salman, A. E. (1987). The use of marine yeast (*Candida sp*.) and bakers’ yeast (*Saccharomyces cerevisiae*) in combination with *chlorella sp*. for mass culture of the rotifer *Brachionus plicatilis* . Hydrobiologia, 147(1), 263–268. 10.1007/BF00025752

[ece39533-bib-0024] Janicke, T. , & Chapuis, E. (2016). Condition dependence of male and female reproductive success: Insights from a simultaneous hermaphrodite. Ecology and Evolution, 6(3), 830–841. 10.1002/ece3.1916 26865970 PMC4739575

[ece39533-bib-0025] Janicke, T. , Chapuis, E. , Meconcelli, S. , Bonel, N. , Delahaie, B. , & David, P. (2022). Environmental effects on the genetic architecture of fitness components in a simultaneous hermaphrodite. Journal of Animal Ecology, 91(1), 124–137. 10.1111/1365-2656.13607 34652857

[ece39533-bib-0026] Janicke, T. , David, P. , & Chapuis, E. (2015). Environment‐dependent sexual selection: Bateman's parameters under varying levels of food availability. The American Naturalist, 185(6), 756–768. 10.1086/681128 25996861

[ece39533-bib-0027] Janicke, T. , Häderer, I. K. , Lajeunesse, M. J. , & Anthes, N. (2016). Darwinian sex roles confirmed across the animal kingdom. Science Advances, 12(2), e1500983. 10.1126/sciadv.1500983 PMC4758741 26933680

[ece39533-bib-0028] Jones, A. G. (2009). On the opportunity for sexual selection, the Bateman gradient and the maximum intensity of sexual selection. Evolution, 63(7), 1673–1684. 10.1111/j.1558-5646.2009.00664.x 19228185

[ece39533-bib-0029] Katsuki, M. , Okada, Y. , & Okada, K. (2012). Impacts of diet quality on life‐history and reproductive traits in male and female armed beetle, *Gnatocerus cornutus* . Ecological Entomology, 37(6), 463–470. 10.1111/j.1365-2311.2012.01390.x

[ece39533-bib-0030] Kelly, C. D. , Stoehr, A. M. , Nunn, C. , Smyth, K. N. , & Prokop, Z. M. (2018). Sexual dimorphism in immunity across animals: A meta‐analysis. Ecology Letters, 21(12), 1885–1894. 10.1111/ele.13164 30288910

[ece39533-bib-0031] Kumar, P. , Yadava, R. K. , Gollen, B. , Kumar, S. , Verma, R. K. , & Yadav, S. (2011). Nutritional contents and medicinal properties of wheat: A review. Life Sciences and Medicine Research, 22(1), 1–10.

[ece39533-bib-0032] Likes, R. , Madl, R. L. , Zeisel, S. H. , & Craig, S. A. (2007). The betaine and choline content of a whole wheat flour compared to other mill streams. Journal of Cereal Science, 46(1), 93–95. 10.1016/j.jcs.2006.11.002.The 19030121 PMC2585782

[ece39533-bib-0033] Maklakov, A. A. , Simpson, S. J. , Zajitschek, F. , Hall, M. D. , Dessmann, J. , Clissold, F. , Raubenheimer, D. , Bonduriansky, R. , & Brooks, R. C. (2008). Sex‐specific fitness effects of nutrient intake on reproduction and lifespan. Current Biology, 18(14), 1062–1066. 10.1016/j.cub.2008.06.059 18635354

[ece39533-bib-0034] Martinossi‐Allibert, I. , Arnqvist, G. , & Berger, D. (2017). Sex‐specific selection under environmental stress in seed beetles. Journal of Evolutionary Biology, 30(1), 161–173. 10.1111/jeb.12996 27749005

[ece39533-bib-0035] Martinossi‐Allibert, I. , Thilliez, E. , Arnqvist, G. , & Berger, D. (2019). Sexual selection, environmental robustness, and evolutionary demography of maladapted populations: A test using experimental evolution in seed beetles. Evolutionary Applications, 12(7), 1371–1384. 10.1111/eva.12758 31417621 PMC6691221

[ece39533-bib-0036] Moiron, M. , Winkler, L. , Martin, O. Y. , & Janicke, T. (2022). Sexual selection moderates heat stress response in males and females. Functional Ecology, 1–11. 10.1111/1365-2435.14204 PMC10092254 37064077

[ece39533-bib-0037] Moorad, J. A. , & Wade, M. J. (2013). Selection gradients, the opportunity for selection, and the coefficient of determination. The American Naturalist, 181(3), 291–300. 10.1086/669158 PMC3620722 23448880

[ece39533-bib-0038] Morimoto, J. , Pizzari, T. , & Wigby, S. (2016). Developmental environment effects on sexual selection in male and female *Drosophila melanogaster* . PLoS One, 11(5), 1–27. 10.1371/journal.pone.0154468 PMC4864243 27167120

[ece39533-bib-0039] Naya, D. E. , Lardies, M. A. , & Bozinovic, F. (2007). The effect of diet quality on physiological and life‐history traits in the harvestman *Pachylus paessleri* . Journal of Insect Physiology, 53(2), 132–138. 10.1016/j.jinsphys.2006.11.004 17196974

[ece39533-bib-0040] Ortuño, J. , Cuesta, A. , Rodríguez, A. , Esteban, M. A. , & Meseguer, J. (2002). Oral administration of yeast, *Saccharomyces cerevisiae*, enhances the cellular innate immune response of gilthead seabream (*Sparus aurata L*.). Veterinary Immunology and Immunopathology, 85(1–2), 41–50. 10.1016/S0165-2427(01)00406-8 11867166

[ece39533-bib-0041] Paaby, A. B. , & Rockman, M. V. (2014). Cryptic genetic variation: Evolution's hidden substrate. Nature Reviews Genetics, 15(4), 247–258. 10.1038/nrg3688 PMC4737706 24614309

[ece39533-bib-0042] Park, T. (1934). Observations on the general biology of the flour beetle, *Tribolium confusum* . The Quarterly Review of Biology, 9(1), 36–54.

[ece39533-bib-0043] Plesnar‐Bielak, A. , Woch, K. R. , Małszycki, M. A. , Alkhawlany, A. T. H. , Hołysz, A. , Assis Correia, J. F. , Turk, N. , Ugrin, M. , Kramarz, P. , & Prokop, Z. M. (2017). Larval and adult nutrition effects on reproductive traits in the red flour beetle. Journal of Zoology, 302(2), 79–87. 10.1111/jzo.12440

[ece39533-bib-0044] Pointer, M. D. , Gage, M. J. G. , & Spurgin, L. G. (2021). *Tribolium* beetles as a model system in evolution and ecology. Heredity, 126, 869–883. 10.1038/s41437-021-00420-1 33767370 PMC8178323

[ece39533-bib-0045] R Core Team . (2018). R: A language and environment for statistical computing. R Foundation for Statistical Computing.

[ece39533-bib-0046] Reddiex, A. J. , Gosden, T. P. , Bonduriansky, R. , & Chenoweth, S. F. (2013). Sex‐specific fitness consequences of nutrient intake and the evolvability of diet preferences. The American Naturalist, 182(1), 91–102. 10.1086/670649 23778229

[ece39533-bib-0047] Riegl, B. , Cavalcante, G. , Bauman, A. G. , Feary, D. A. , Steiner, S. , & Purkis, S. (2017). Demographic mechanisms of reef coral species winnowing from communities under increased environmental stress. Frontiers in Marine Science, 4, 1–16. 10.3389/fmars.2017.00344

[ece39533-bib-0048] Rowe, L. , & Houle, D. (1996). The lek paradox and the capture of genetic variance by condition dependent traits. Proceedings of the Royal Society B: Biological Sciences, 263(1375), 1415–1421. 10.1098/rspb.1996.0207

[ece39533-bib-0049] Sakai, M. , Taniguchi, K. , Mamoto, K. , Ogawa, H. , & Tabata, M. (2001). Immunostimulant effects of nucleotide isolated from yeast RNA on carp, *Cyprinus carpio L* . Journal of Fish Diseases, 24(8), 433–438. 10.1046/j.1365-2761.2001.00314.x

[ece39533-bib-0050] Sbilordo, S. H. , & Martin, O. Y. (2014). Pre‐ and postcopulatory sexual selection act in concert to determine male reproductive success in *Tribolium castaneum* . Biological Journal of the Linnean Society, 112(1), 67–75. 10.1111/bij.12262

[ece39533-bib-0051] Schmidt, G. , Seraidarian, K. , Greenbaum, L. M. , Hickey, M. D. , & Thannhauser, S. J. (1956). The effects of certain nutritional conditions on the formation of purines and of ribonucleic acid in baker's yeast. Biochimica et Biophysica Acta ‐ General Subjects, 20(C), 135–149. 10.1016/0006-3002(56)90272-4 13315360

[ece39533-bib-0052] Siwicki, A. K. , Anderson, D. P. , & Rumsey, G. L. (1994). Dietary intake of immunostimulants by rainbow trout affects non‐specific immunity and protection against furunculosis. Veterinary Immunology and Immunopathology, 41(1–2), 125–139. 10.1016/0165-2427(94)90062-0 8066989

[ece39533-bib-0053] Sokoloff, A. , Franklin, I. R. , Overton, L. F. , & Ho, F. K. (1966). Comparative studies with *Tribolium* (coleoptera, Tenebrionidae) ‐I: Productivity of *T. castaneum* (Herbst) and *T. confusum Duv*. On several commercially available diets. Journal of Stored Products Research, 1, 295–311.

[ece39533-bib-0054] Sommer, S. , Piscia, R. , Manca, M. M. , Fontaneto, D. , & Ozgul, A. (2016). Demographic cost and mechanisms of adaptation to environmental stress in resurrected *daphnia* . Journal of Limnology, 75(2S), 30–35. 10.4081/jlimnol.2016.1292

[ece39533-bib-0055] Than, A. T. , Ponton, F. , & Morimoto, J. (2020). Integrative developmental ecology: A review of density‐dependent effects on life‐history traits and host‐microbe interactions in non‐social holometabolous insects. Evolutionary Ecology, 34(5), 659–680. 10.1007/s10682-020-10073-x

[ece39533-bib-0056] Travers, L. M. , Garcia‐Gonzalez, F. , & Simmons, L. W. (2015). Live fast die young life history in females: Evolutionary trade‐off between early life mating and lifespan in female *Drosophila melanogaster* . Scientific Reports, 5, 1–7. 10.1038/srep15469 PMC4612512 26482533

[ece39533-bib-0057] Viña, J. , Borrás, C. , Gambini, J. , Sastre, J. , & Pallardó, F. V. (2005). Why females live longer than males? Importance of the upregulation of longevity‐associated genes by oestrogenic compounds. FEBS Letters, 579(12), 2541–2545. 10.1016/j.febslet.2005.03.090 15862287

[ece39533-bib-0058] Vinogradov, A. E. (1998). Male reproductive strategy and decreased longevity. Acta Biotheoretica, 46, 157–160. 9691260 10.1023/a:1001181921303

[ece39533-bib-0059] Wade, M. J. , & Shuster, S. M. (2005). Don't throw Bateman out with the bathwater! Integrative and Comparative Biology, 45(5), 945–951. 10.1093/icb/45.5.945 21676845

[ece39533-bib-0060] Whitlock, M. C. , & Agrawal, A. F. (2009). Purging the genome with sexual selection: Reducing mutation load through selection on males. Evolution, 63(3), 569–582. 10.1111/j.1558-5646.2008.00558.x 19154364

[ece39533-bib-0061] Wickham, H. (2016). ggplot2: Elegant graphics for data analysis. Springer‐Verlag.

[ece39533-bib-0062] Wilder, S. M. , Raubenheimer, D. , & Simpson, S. J. (2016). Moving beyond body condition indices as an estimate of fitness in ecological and evolutionary studies. Functional Ecology, 30(1), 108–115. 10.1111/1365-2435.12460

[ece39533-bib-0063] Willi, Y. , & Hoffmann, A. A. (2009). Demographic factors and genetic variation influence population persistence under environmental change. Journal of Evolutionary Biology, 22(1), 124–133. 10.1111/j.1420-9101.2008.01631.x 19120814

[ece39533-bib-0067] Winkler, L. , & Janicke, T. (2022). Diet quality impairs male and female reproductive performance and affects the opportunity for selection in an insect model [Data set]. Dryad. 10.5061/DRYAD.05QFTTF6H PMC9682208 36440316

[ece39533-bib-0064] Winkler, L. , Moiron, M. , Morrow, E. H. , & Janicke, T. (2021). Stronger net selection on males across animals. eLife, 10, e68316. 10.7554/eLife.68316 34787569 PMC8598160

[ece39533-bib-0065] Wong, N. , & Lee, C. Y. (2011). Relationship between population growth of the red flour beetle *Tribolium castaneum* and protein and carbohydrate content in flour and starch. Journal of Economic Entomology, 104(6), 2087–2094. 10.1603/EC11234 22299375

[ece39533-bib-0066] Zikovitz, A. E. , & Agrawal, A. F. (2013). The condition dependency of fitness in males and females: The fitness consequences of juvenile diet assessed in environments differing in key adult resources. Evolution, 67(10), 2849–2860. 10.1111/evo.12170 24094338

